# Organophosphates and Outdoor Air: Harnly et al. Respond

**Published:** 2006-06

**Authors:** Martha Harnly, Robert McLaughlin, Robert Gunier, Asa Bradman, Meredith Anderson

**Affiliations:** California Department of Health Services, Richmond, California, E-mail: mharnly@dhs.ca.go; University of California, Berkeley, CA; Impact Assessment Inc., Richmond, CA

Peterson is incorrect in stating that we did not conduct a risk assessment. We summarized in our article (Harnly et al. 2005) and detailed in a previous article (Lee et al. 2002) a human health risk assessment demonstrating elevated acute and sub-chronic risks for children’s exposures to ambient chlorpyrifos air concentrations in agricultural communities. Compared with the assessment by Peterson, we used a more refined probability distribution analysis, included air levels of the degradation product (chlorpyrifos oxon), and presented exposures relative to reference values. The chlorpyrifos reference values, however, were based on the same no-observed-adverse-effect levels (NOAELs) and the same 10-fold intraspecies and interspecies, and child uncertainty factors used in Peterson’s calculations.

In our article (Harnly et al. 2005), we suggested that our risk assessment may underestimate risks for several reasons, such as *a*) risk assessments that use NOAELs, and not the entire dose–response curve, tend to underestimate risks (Castorina and Woodruff, 2003); and *b*) the true ranges of intraspecies and interspecies variability are unknown and may be larger than the factors used (Eskenazi et al. 1999; Faustmann et al. 2000). In a very recent study, some newborns were 65–130 times less able to metabolize diazoxon and chlorpyrifos oxon than their mothers (Furlong et al. 2006). To further support the concern for children indicated by our quantitative risk assessment, we cited toxicologic studies establishing that in addition to chloinesterase inhibition, on which the NOAEL for chlorpyrifos is established, chlorpyrifos and chlorpyrifos oxon have other neurodevelopmental toxicity mechanisms (Huff et al. 1994; Qiao et al. 2002). We also noted that cell death has been induced at the reference dose for drinking water (Greenlee et al. 2005).

Peterson argues that the toxicologic studies we cited (Castorina and Woodruff, 2003; Eskenazi et al. 1999; Faustmann et al. 2000; Greenlee et al. 2005; Huff et al. 1994; Qiao et al. 2002) are an insufficient review of the “literature relevant to risk assessment” and that these studies are not appropriate for use in risk assessment. However, in missing the fact that we conducted a quantitative risk assessment, Peterson is misinterpreting our citations as the only basis for our public health concern. We consider it our public health responsibility to at least qualitatively consider recent toxicologic data in addition to a quantitative risk assessment based on established reference values. Others have argued for a complete restructuring of risk assessment for children, including toxicokinetic modeling and assessment of cellular and molecular outcomes over the entire lifespan of experimental subjects (Landrigan et al. 2004).

For many reasons we disagree with the suggestion that the epidemiologic fetal growth and gestational duration findings of Eskenazi et al. (2004) may be used to disregard concern for *in utero* and child organophosphate exposure highlighted by Eskenazi et al. (1999). The associations of reduced gestational duration with dimethyl organophosphate urinary metabolites and chloinesterase inhibition were not clinically significant in the California population studied (recent Mexican immigrants who tend to have very healthy birth outcomes). However, a shortened gestational age of a half-week would represent, for some women, a risk of preterm delivery (Eskenazi et al. 2004). Clearly, this finding and the absence of any adverse association between fetal growth and measures of *in utero* pesticide exposure need to be confirmed or refuted. To be complete, however, we also cited the association found in a New York City population between low birth weight and length and cord plasma levels of chlorpyrifos and diazinon (*n* = 314) (Whyatt et al. 2004). Further, effects of organophosphate pesticide exposure on early child neurodevelopment have been found (Young et al. 2005) and are continuing to be evaluated in the California and the New York City cohorts. Finally, public health policy is typically developed to protect against a 1 in 1,000, or lower, risk, and the epidemiologic studies cited here are below the sample size necessary to detect such risks.

Peterson notes that a study of children in 10 homes did not demonstrate an association with child urine metabolite levels of chlorpyrifos and ambient air levels following crack and crevice treatment (Hore et al. 2005). Yet, the authors of that study were careful to note a number of study limitations, including the variability and accuracy of the child urinary metabolite readings. We also note that chloryprifos oxon, which also breaks down into the measured urinary metabolite, was not measured in air; air concentrations in four of the study homes were not elevated compared to pretreatment levels; and personal air samples were not collected (Hore et al. 2005). Among mothers in New York City (*n* = 314) in another study, 48-hr personal air samples collected during pregnancy were associated with cord and maternal blood levels of chlorpyrifos (Whyatt et al. 2004). This is the same study population within which an association with adverse birth outcomes and pesticide cord blood levels has been demonstrated, and the chlorpyrifos air levels are in the same (average, 15 ng/m^3^) range, if not lower, as those evaluated in our health risk assessment (Whyatt et al. 2004).

## References

[b1-ehp0114-a00339] Castorina R, Woodruff TJ (2003). Assessment of potential risk levels associated with U.S. Environmental Protection Agency reference values. Environ Health Perspect.

[b2-ehp0114-a00339] Eskenazi B, Bradman A, Castorina R (1999). Exposures of children to organophosphate pesticides and their potential health effects. Environ Health Perspect.

[b3-ehp0114-a00339] Eskenazi B, Harley K, Bradman A, Weltzien E, Jewell NP, Barr DB (2004). Association of *in utero* organophosphate pesticide exposure and fetal growth and length of gestation in an agricultural population. Environ Health Perspect.

[b4-ehp0114-a00339] Faustman EM, Silbernagel SM, Fenske RA, Burbacher TM, Ponce RA (2000). Mechanisms underlying children’s susceptibility to environmental toxicants. Environ Health Perspect.

[b5-ehp0114-a00339] Furlong CE, Holland N, Richter RJ, Bradman A, Ho A, Eskenazi B (2006). PON1 status of farmworker mothers and children as a predictor of organophosphate sensitivity. Pharmacogenet Genomics.

[b6-ehp0114-a00339] Greenlee AR, Ellis TM, Berg RL (2004). Low-dose agrochemicals and lawn-care pesticides induce developmental toxicity in murine preimplantation embryos. Environ Health Perspect.

[b7-ehp0114-a00339] Harnly M, McLaughlin R, Bradman A, Anderson M, Gunier R (2005). Correlating agricultural use of organophosphates with outdoor air concentrations: a particular concern for children. Environ Health Perspect.

[b8-ehp0114-a00339] Hore P, Robson M, Freeman N, Zhang J, Wartenberg D, Ozkaynak H (2005). Chlorpyrifos accumulation patterns for child-accessible surfaces and objects and urinary metabolite excretion by children for 2 weeks after crack-and-crevice application. Environ Health Perspect.

[b9-ehp0114-a00339] Huff RA, Corcoran JJ, Anderson JK, Abou-Donia MB (1994). Chlorpyrifos oxon binds directly to muscarinic receptors and inhibits cAMP accumulation in rat striatum. J Pharmacol Exp Ther.

[b10-ehp0114-a00339] Landrigan PJ, Kimmel CA, Correa A, Eskenazi B (2004). Children’s health and the environment: public health issues and challenges for risk assessment. Environ Health Perspect.

[b11-ehp0114-a00339] Lee S, McLaughlin R, Harnly M, Gunier R, Kreutzer R (2002). Community exposures to airborne agricultural pesticides in California: ranking of inhalation risks. Environ Health Perspect.

[b12-ehp0114-a00339] Qiao D, Seidler FJ, Padilla S, Slotkin TA (2002). Developmental neurotoxicity of chlorpyrifos: what is the vulnerable period?. Environ Health Perspect.

[b13-ehp0114-a00339] Whyatt RM, Rauh V, Barr DB, Camann DE, Andrews HF, Garfinkel R (2004). Prenatal insecticide exposures and birth weight and length among an urban minority cohort. Environ Health Perspect.

[b14-ehp0114-a00339] Young JG, Eskenazi B, Gladstone EA, Bradman A, Pedersen L, Johnson C (2005). Association between in utero organophosphate pesticide exposure and abnormal reflexes in neonates. Neurotoxicology.

